# Investigation of Anthocyanins Stability from Pomegranate Juice (*Punica Granatum* L. Cv Ermioni) under a Simulated Digestion Process

**DOI:** 10.3390/medicines6030090

**Published:** 2019-08-20

**Authors:** Chrysavgi Gardeli, Kalliopi Varela, Eleni Krokida, Athanasios Mallouchos

**Affiliations:** Department of Food Science and Human Nutrition, Agricultural University of Athens, Iera Odos 75, 11855 Athens, Greece

**Keywords:** anthocyanins, pomegranate juice, simulated digestion process, phenolics, antioxidant activity

## Abstract

**Background:** Pomegranate gained a widespread popularity as a functional food due to the high content of bioactive components of the whole fruit, as well as its juice and extracts. There is a large amount of research that assigns them very important functions for the human organism. **Methods:** The anthocyanins (ACNs) of pomegranate juice (PJ) from the Ermioni variety are quantitatively identified and their stability under a simulated digestion process (SDP) is investigated. ACNs, as well as phenolic compounds, were isolated through solid phase extraction and determined using high-performance liquid chromatography in every stage of the SDP. Total phenolics, total monomeric ACNs, polymeric color and antioxidant activity were also determined in pomegranate juice and during the digestion process. **Results:** The predominant anthocyanin was Cy-3-glucoside followed by the corresponding 3,5-diglucoside, which accounted for 40.8% and 27.4% of the total ACN content, respectively. About 65% of the total monomeric ACN content remained intact by the end of the simulated digestion process. **Conclusions:** The PJ of the Ermioni variety seems to retain a large amount of the bioactive compounds after the SDP. The antioxidant activity and total phenolic content (TPC) remain almost stable during the SDP, suggesting that the products formed during ACN degradation maintain the antioxidant activity of the parent molecule.

## 1. Introduction

It is widely accepted that fruit and vegetable consumption has beneficial health effects associated with disease prevention due to their high content of bioactive components. Red fruits, such as strawberries, cherries, grapes and pomegranates, have been characterized as such sources because they are rich in phenolic compounds such as phenolic acids, flavonoids and tannins [1]. In particular, pomegranate has gained widespread popularity as a functional food due to the high content of bioactive components of the whole fruit, as well as its juice and extracts. There is a large amount of research that assigns them very important functions for the human organism [2]. These components possess antimicrobial and anti-inflammatory properties [3], can act as antioxidants [4], anticancer agents [5], antihepatotoxic agents [6] and can improve cardiovascular [7], oral [8] and skin health [9].

Anthocyanins (ACNs) are the largest and most important group of flavonoids present in pomegranate juice (PJ) and together with hydrolysable tannins (HTs) they constitute the most valuable bioactive compounds. Chemically, ACNs are glycosides of polyhydroxy and polymethoxy derivatives of 2-phenylbenzopyrylium or flavylium salts (2 benzoyl rings, A and B, are separated by a heterocyclic C- ring) [10,11]. Although it is possible to find hundreds of different ACNs due to complex glycosylation patterns, there are only a few aglycones (called anthocyanidins) associated with their structure. Upon hydrolysis, the ACNs’ glycosidic bond breaks to free the sugar molecule and the aglycon. The high in vitro antioxidant activity of pomegranate has been largely attributed to its ellagitannin content [12], which belongs to the HTs class and, upon hydrolysis, releases ellagic acid.

Among the 25 different aglycones that have been identified, only 6 anthocyanidins, namely cyanidin (Cy), delphinidin (Dp), malvidin (Mv), pelargonidin (Pg), peonidin (Pn), and petunidin (Pt), are widely distributed in nature, accounting for over 90% of the ACNs identified [10,13]. PJ is characterized by the presence of the 3-glucosides and 3,5-diglucosides of Dp, Cy, and Pg [14]. The free radical scavenging activity of these compounds has been demonstrated in various studies, such as that conducted by Espín et al. (2000) [15]. An estimated 10% of the antioxidant action of PJ is due to the presence of ACNs [16].

Compared to the other flavonoids, they can be absorbed as intact molecules and circulate in the plasma and pass into urine without undergoing metabolic changes [17,18]. The major absorbed and excreted components are not only affected by the structure of the ACN aglycon but also by the nature of the sugar moiety [19,20]. Moreover, ACNs, unlike other flavonoids, are characterized by rearrangements in response to pH changes, which means that they are more stable at the acidic pH of the stomach, where they exist in the form of red flavylium cation. Under basic conditions (pH > 7), such as those that occur in the small intestine, ACNs are in the form of colorless carbinol, which undergoes limited absorption and is probably metabolized into conjugates that are overlooked, since they cannot be converted to flavylium forms [18,21].

Once ACNs are absorbed, they could be readily distributed to different organs, including the liver, lung, kidney, prostate, heart, and brain [11,22,23]. There is evidence that the ACNs’ transportation mechanism into cells and tissues depends on the number and the position of the hydroxyl groups in their structure. Therefore, studying the composition of food ACNs and their metabolic products during in vitro digestion may help with evaluating their in vivo forms and better understanding their bioactivity [21]. Concerning the in vitro digestion experiments, there are studies on different parts of the whole fruit, such as the juice [24,25] (cv Mollar), pulp, peel extracts, peel flour and commercial fruit juice [26,27,28], available in the literature. In our work, a simulated digestion process (SDP) was applied to PJ of the Ermioni variety to investigate its ACNs’ bioavailability and to provide data for future in vivo experiments.

The Ermioni variety (its name comes from the homonymous growing region in the Peloponnese), is of great economic interest for Greece. Its cultivation has increased greatly over the last few years—from 4000 acres in 2007 to 10,000 in 2014—and has expanded into many areas of central and northern Greece. In the present study, the ACNs of PJ from the Ermioni variety were determined quantitatively and their stability under the SDP was investigated. The changes in the monomeric and the polymeric ACN content of PJ as a function of pH and temperature conditions was also investigated, together with the antioxidant activity and the total phenolic content through the SDP. The PJ of the Ermioni variety seemed to retain a large amount of its bioactive compounds after the SDP. The antioxidant activity and total phenolic content (TPC) remained almost constant during the SDP suggesting that the products formed during ACN degradation maintained the antioxidant activity of the parent molecule. To the best of our knowledge, this is the first study reporting the chemical characteristics of the ACNs of PJ from the Ermioni variety. Our findings could provide useful information for the elucidation of the pomegranate ACNs’ stability in the human gastrointestinal tract and could boost the utilization of PJ as a functional food.

## 2. Materials and Methods

### 2.1. Chemicals and Reagents

Cyanidin chloride was purchased from Extrasynthese (Genay, France); Folin-Ciocalteu reagent, DPPH (2,2-diphenyl-1-picrylhydrazyl), formic acid and gallic acid were purchased from Sigma-Aldrich (St. Louis, MO, USA). High performance liquid chromatography (HPLC) grade water and acetonitrile were purchased from Honeywell (Seelze, Germany). All other reagents were of analytical grade and were obtained from Fluka (Buchs, Germany) or Merck (Darmstadt, Germany).

### 2.2. Juice Preparation

Approximately 4 kg of pomegranate fruits (*Punica granatum* L., Greek variety Ermioni), region of origin Aigio (Peloponnese) were purchased from the local market. After discarding injured and sunburnt fruits, they were peeled and the skins covering the seeds were removed manually. The juicy sacs from the fruit pericarp were separated by hand, placed in a muslin cloth and hand-pressed. The juice was covered with tin foil to avoid light and it was kept at −80 °C until analyzed. Before the experiments, the PJ was defrosted and then centrifuged at 1792 RCF for 15 min in order to remove water insoluble particles.

### 2.3. Simulated Digestion Process

Simulated gastrointestinal digestion was carried out according to Yang et al. (2018) [29] with minor modifications. The process involved two consecutive stages in specific pH and temperature conditions in order to mimic gastric and intestinal digestion. For the first stage (gastric digestion), 180 mL of juice sample was adjusted to pH 1.5 using a concentrated HCl solution. Subsequently, the juice sample was incubated at 37 °C for 6 h under constant shaking at 200 rpm. During digestion, samples were taken every 2 h and kept at −80 °C until further analysis. For the second stage (intestinal digestion), the remaining juice sample was immediately adjusted to pH 7.5 using a NaOH solution (4 M) and incubated under the same conditions for another 6 h. Sampling was accomplished as previously described. Thus, for the whole 12 h-digestion period, a series of 6 juice samples plus the initial one (non-incubated) were taken and labelled as follows—0h (initial juice adjusted at pH 1.5 without incubation), G-2 h, G-4 h, G-6 h as treatments after 2, 4 and 6 h in simulated gastric conditions, and I-2 h, I-4 h and I-6 h as treatments after 2, 4, 6 h in simulated intestinal conditions. The described procedure was carried out three times.

### 2.4. Determination of Total Monomeric Anthocyanin Content

The total monomeric ACN content was determined using the pH-differential method described previously [30]. For each test sample, two dilutions were prepared; one with potassium chloride buffer (pH 1.0) and the other with sodium acetate buffer (pH 4.5). These were left to equilibrate for 15 min at room temperature and then the absorbance was read at 510 nm (λ_max_) for ACN content and at 700 nm for haze correction. All absorbance measurements were carried out on a UV-Vis double-beam spectrophotometer (Jasco V-530, Tokyo, Japan) using 1 cm path length disposable cuvettes against distilled water as a blank and repeated three times. The absorbance (A) of the diluted juice samples was calculated from Equation (1):

A = (A_510 nm_ − A_700 nm_)_pH 1.0_ − (A_510 nm_ − A_700 nm_)_pH 4.5_ (1)

The total monomeric ACN content was expressed as cyanidin-3-glucoside (Cy-3-glu) equivalents (mg/L) and calculated by Equation (2):

Cyanidin-3-glucoside eq., mg/L = (A × *M* × DF × 1000)/(ε × l) (2)

where A = absorbance value from Equation (1), *M* = molar mass of Cy-3-glu (449.2 g/mol), DF = dilution factor used (20), ε: molar absorptivity of Cy-3-glu (26,900 L cm^−1^ mol^−1^), l = path length (in cm) and 1000 = conversion factor of g to mg.

### 2.5. Determination of Polymeric Color

Polymeric color content was determined using the bisulfite bleaching method as described previously [30]. The test sample was diluted appropriately with 0.1 M citric acid buffer (pH 3.5). Then, 2.8 mL of diluted sample was transferred to each of two disposable cuvettes and 0.2 mL of bisulfite solution was added to one cuvette, whereas 0.2 mL of distilled water was added to the other. The solutions were left to equilibrate for 15 min at room temperature. The absorbance was read at 420 nm for brown pigments, at 510 nm for monomeric ACNs and at 700 nm for haze correction. All absorbance measurements were carried out on a UV-Vis double-beam spectrophotometer (Jasco V-530) using 1 cm path length disposable cuvettes against distilled water as a blank and repeated three times. The color density of the control sample (water treated) was calculated using Equation (3):

Color density = [(A_420 nm_ − A_700 nm_) + (A_510 nm_ − A_700 nm_)] × DF (3)

where: DF = dilution factor used = 20.

The polymeric color of the bisulfite treated sample was calculated using Equation (4):

Polymeric color = [(A_420 nm_ − A_700 nm_) + (A_510 nm_ − A_700 nm_)] × DF (4)

The percent polymeric color was calculated by Equation (5):

% polymeric color = (polymeric color/color density) × 100 (5)

### 2.6. Determination of Total Phenolic Content

TPC was determined using the Folin-Ciocalteu method [31]. One mL of appropriately diluted sample (2.5% *v*/*v* in water) and 5 mL of 10-fold diluted Folin-Ciocalteu reagent were added to a screw-capped glass vial. After a period of 3–8 min, 4 mL of 7.5% (*w*/*v*) sodium carbonate solution was added, the content was mixed and left in the dark for two hours at room temperature. As a blank sample, 1 mL of deionized water was used and treated as previously. The absorbance was measured at 765 nm on a UV-Vis double-beam spectrophotometer (Jasco V-530). Two measurements were performed for each test sample. The same procedure was applied for six standard solutions of gallic acid (5–50 μg/mL) and a calibration curve was generated. Final results were expressed as gallic acid equivalents (GAE) mg/L.

### 2.7. Determination of Antioxidant Activity

Antioxidant activity was measured using the stable free radical DPPH as described by Molyneux P. (2004) [32]. Briefly, a series of dilutions of each test sample was made using water/methanol 60:40 *v*/*v*. Then, 2 mL of each dilution were mixed with 2 mL of DPPH working solution (0.2 μM in methanol) in a screw-capped glass tube. The tube was vortexed and left in the dark for 30 min at room temperature. The absorbance was measured at 517 nm against methanol as a blank. As a control sample, 2 mL of the dilution solvent mixture was used and treated as described previously. The radical scavenging activity (RSA%) was estimated using Equation (6):

(6) $$\mathrm{RSA}\%=\frac{A_{\mathrm{control}}-A_{\mathrm{sample}}}{A_{\mathrm{control}}}\times 100$$

For the interpretation of the results, the parameter of “Efficient Concentration” or EC_50_ value (defined as the concentration of substrate that causes 50% loss of the DPPH activity) was used. It was calculated by interpolation from the graph of RSA% against the sample concentration (in cuvette).

### 2.8. Solid Phase Extraction of Phenolic Compounds and High Performance Liquid Chromatography

Isolation of ACNs and non-ACNs phenolic fractions of PJ and all series of digestion samples were carried out using Solid Phase Extraction (SPE), as described by Rodriguez-Saona et al. (2004) [30], with minor modifications. All samples were adjusted to pH 2–3 by using either NaOH 4N solution or concentrated HCl solution. Prior to sample loading, the SPE cartridge (C18, 500 mg, Macherey-Nagel, Düren, Germany) was activated with 6 mL acidified methanol (0.01% *v*/*v* HCl), followed by 6 mL acidified distilled water (0.01% *v*/*v* HCl). After loading 4.5 mL of sample, the cartridge was washed with 12 mL acidified water to remove interfering compounds, such as sugars and organic acids, and then it was dried under a stream of N_2_ for 2–3 min. Non-ACNs phenolics were eluted by rinsing the cartridge with 12 mL ethyl acetate. Elution of ACNs was carried out by rinsing the cartridge with acidified methanol. The solvents were removed in a rotary evaporator (IKA RV05, Staufen, Germany) at 40 °C under vacuum. The non-ACNs phenolic fraction was re-dissolved in 2 mL H_2_O:MeOH (95:5 *v*/*v*) acidified with 0.1% *v*/*v* formic acid, whereas the ACNs fraction was re-dissolved in 2 mL H_2_O:MeOH (80:20 *v*/*v*) acidified with 5% *v*/*v* formic acid. The extracts were stored in vials under nitrogen gas and kept at −20 °C until HPLC analysis.

Chromatographic analysis of the ACNs fraction was carried out on a Perkin Elmer Flexar HPLC system equipped with an on-line degasser, a quaternary pump, an auto sampler, a column oven and a photodiode array detector. Separation was performed on a Kinetex EVO C18 column (100 mm × 4.6 mm i.d., 2.6 μm particle size, Phenomenex, Torrance, CA, USA) set at 35 °C using water as mobile phase A and methanol as mobile phase B, both acidified with formic acid 0.1% *v*/*v*. The gradient elution program was the following—80% A from 0 to 1 min, 70% A at 5.5 min, 60% A from 5.6 to 7.6 min. The initial condition was reached in 0.1 min and the column left to equilibrate for 8 min. The flow rate was held constant at 0.8 mL/min and the injection volume was 5 μL. Spectra were recorded from 200 to 600 nm. Due to the unavailability of ACN standards, peak identification was based on the comparison of spectra of unknown compounds with those previously reported in the literature [33]. Peak quantitation was based on an external standard method using the peak area recorded at 520 nm. A calibration curve was constructed using cyanidin chloride as the external standard. All ACNs were expressed as cyanidin chloride equivalents (mg/L).

Chromatographic analysis of the non-ACNs phenolic fraction was carried out on a Jasco HPLC system equipped with a quaternary pump (PU-2089 Plus, Tokyo, Japan), an auto sampler (AS-1555, Tokyo, Japan), a column oven (Jones Chromatography, model 7990, Mid Glamorgan, United Kingdom) and a photodiode array detector (MD-910, Tokyo, Japan). Separation was performed on a Spherisorb ODS2 column (250 mm × 4.6 mm i.d., 5 μm particle size, Waters, Milford, MA, USA) set at 30 °C using a gradient elution with initial composition of 95:5 (*v*/*v*) water/methanol acidified with 0.1% *v*/*v* formic acid (mobile phase A). Methanol acidified with 0.1% *v*/*v* formic acid was used as mobile phase B. The gradient program was the following—100% A at 0 min, 74% A at 15 min, 63% A at 40 min, 53% A at 60 min, 47% A at 65 min, 0% A at 90 min and maintained there for 5 min. The initial condition was reached in 2 min and the column was left to equilibrate for 20 min. The flow rate was held constant at 0.9 mL/min and the injection volume was 20 μL. Spectra were recorded from 200 to 600 nm. Peak identification was based on the comparison of retention times and spectra of unknown compounds with those of standards. Quantitation was based on external standard method using peak area at λ_max_.

### 2.9. Statistical Analysis

The data obtained from replicate determinations were averaged and reported along with standard deviation. Analysis of Variance (ANOVA) and post hoc comparisons using Tukey’s honest significant difference (HSD) test were performed (at *p* < 0.05) to reveal statistical differences among treatments. Statistical analyses were carried out using Statgraphics Centurion XVI (Version 16.1.11, StatPoint Technologies Inc., Warrenton, VA, USA).

## 3. Results

### 3.1. Characterization of Pomegranate Juice from Ermioni Variety

The chemical characteristics of PJ from the Ermioni variety are presented in Table 1. The pH was within the normal range reported in the literature [34]. Its ACNs profile was characterized by the presence of diglucosides and monoglucosides of Dp, Cy and Pg. The predominant ACN was the Cy-3-glucoside followed by the Cy-3,5-diglucoside which accounted for the 40.8% and 27.4% of the total ACN content, respectively. The concentration of ACN monoglucosides (214.2 mg/L) represented the 56.0% of the total ACNs content of the juice, exceeding the content of ACN diglucosides (168.6 mg/L). The total concentration of ACNs, as determined by HPLC after SPE extraction, was found to be approximately two times higher than the total monomeric ACN content, which was determined by the pH-differential method. Similar discrepancies have been observed by other researchers [35]. In most cases, the total monomeric ACN content is reported higher when determined by the pH-differential method than by HPLC. However, Turfan et al. (2011) [35] observed the opposite in juice from pomegranate obtained from sacs, as in the present study. A possible explanation for the observed difference between the two methods can be attributed to the different solvent system utilized that affects the spectral characteristics of ACNs [36].

The polymeric color was equal to 12%, which is consistent with juice freshness. Fresh fruit or vegetable juices have a low percentage of polymeric color (usually less than 10%), while processed samples and materials subjected to storage abuse tend to have higher values (30% or more) [30].

The high TPC value (1271 GAE mg/L) found in the present study lies within the usual range reported by other researchers (651–3098 mg/L) [16,34,37]. The antioxidant activity of Ermioni PJ was equal to 0.35% (*v*/*v*) expressed as EC_50_. Similar results were obtained previously [38].

### 3.2. Evolution of ACNs During the Simulated Digestion Process

**Figure 1:**
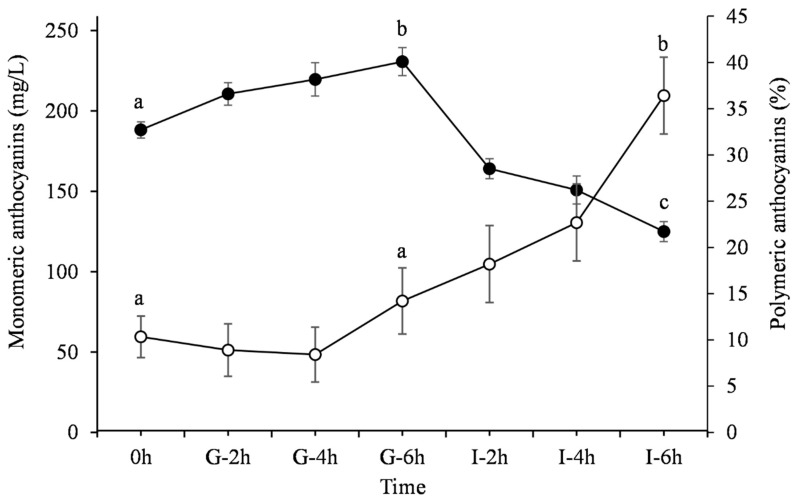
The evolution of the total monomeric anthocyanins (ACNs) content (-●-) and percent polymeric color (-○-) of PJ under simulated digestion process (SDP) (0 h; G-2 h, G-4 h, G-6 h: samples under the gastric condition; I-2h, I-4h, I-6h: samples under the intestinal condition). Different letters denote statistical significance (*p* < 0.05) between time points of each curve.

Figure 1 presents the evolution of the total monomeric ACNs content and percent polymeric color of PJ during the SDP. It was observed that the incubation of PJ during simulated gastric conditions resulted in a 22.5% increase of the total monomeric ACN content after 6 h (*p* <0.001). However, its concentration decreased by about 33.6% after digesting the juice in the simulated intestinal pH conditions (after 12 h) (*p* < 0.001). Our results are in agreement with those of a similar study conducted in wine under similar gastrointestinal pH and temperature conditions [29].

As regards the percent polymeric color, no significant difference (*p* = 0.47) was observed during the stage of gastric digestion (Figure 1). It is evident, though, that it increased significantly throughout the intestinal phase (*p* < 0.001).

**Figure 2:**
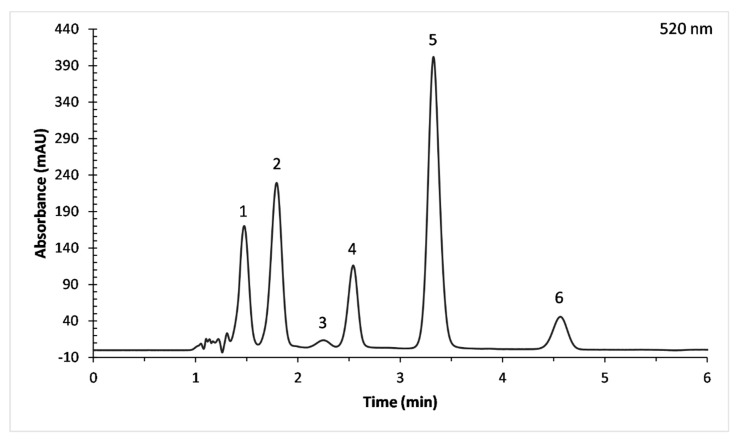
High performance liquid chromatography (HPLC) chromatogram at 520 nm of PJ (Ermioni variety) ACNs. 1: Dp-3,5-diglucoside, 2: Cy-3,5-diglucoside, 3: Pg-3,5-diglucoside, 4: Dp-3-glucoside, 5: Cy-3-glucoside and 6: Pg-3-glucoside.

**Figure 3:**
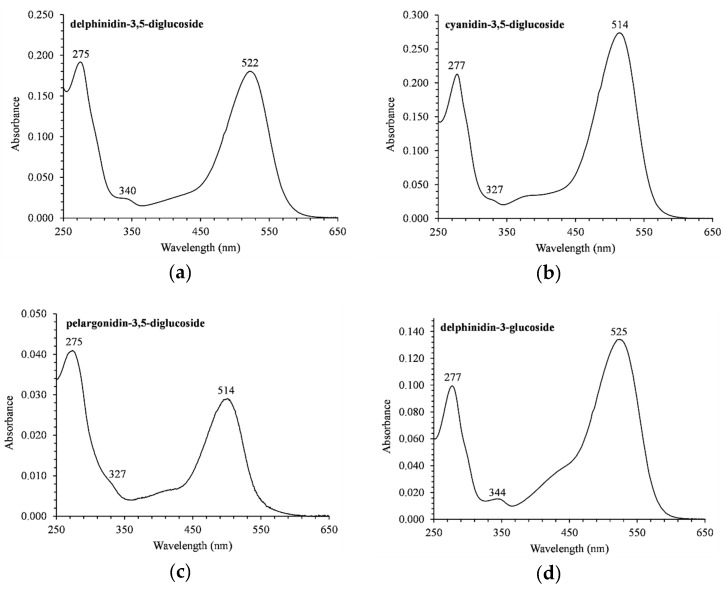

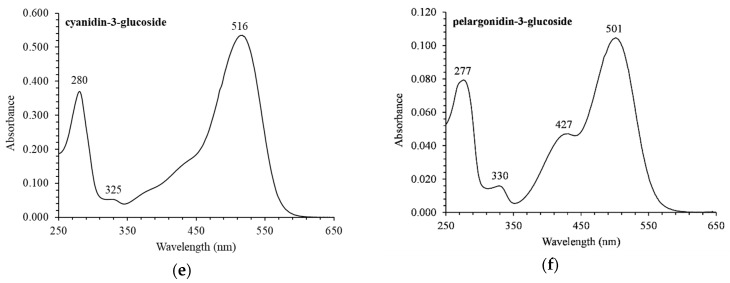
UV-Vis spectra of the ACNs found in PJ from the Ermioni variety: (**a**) Dp-3,5-diglucoside; (**b**) Cy-3,5-diglucoside; (**c**) Pg-3,5-diglucoside; (**d**) Dp-3-glucoside; (**e**) Cy-3-glucoside; (**f**) pelargonidin-3-glucoside.

Figure 2 presents a typical HPLC chromatographic separation of the ACNs fraction of PJ from the Ermioni variety. Six different ACNs were identified according to the elution order and spectral characteristics from the literature due to the unavailability of authentic compounds. They had a typical absorption band in the 500–525 nm region of the visible spectra (Figure 3). Another distinctive band was observed in the UV-region (275–280 nm).

**Figure 4:**
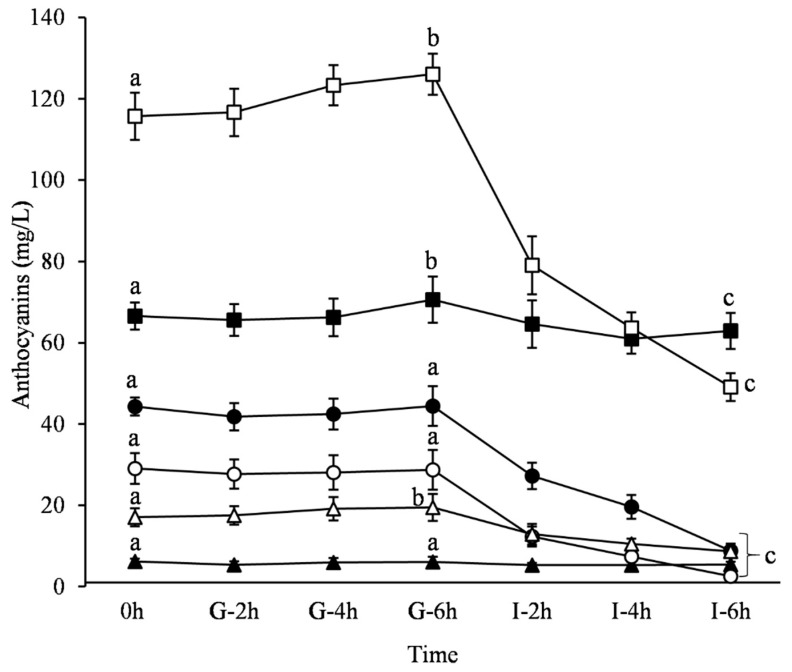
Evolution of each individual ACN of PJ during the simulated digestion process (SDP) (0 h; G-2 h, G-4 h, G-6 h: samples under the gastric condition; I-2 h, I-4 h, I-6 h: samples under the intestinal condition; -●-: Dp-3,5-diglucoside; -○-: Dp-3-glucoside; -■-: Cy-3,5-diglucoside; -□-: Cy-3-glucoside; -▲-: Pg-3,5diglucoside; -∆-: Pg-3-glucoside). Different letters denote statistical significance (*p* < 0.05) between time points of each ACN.

During the simulated gastric phase, the concentration of Dp-3,5-diglucoside, Pg-3,5-diglucoside and Dp-3-glucoside remained practically constant (*p* > 0.05) (Figure 4). On the contrary, the content of Cy-3,5-diglucoside, Cy-3-glucoside and Pg-3-glucoside increased during the last 2h of the gastric phase (*p* < 0.05) by 6%, 9% and 14%, respectively. This could be attributed to the highly acidic environment (pH 1.5), which favors the prevalence of the flavylium cation conformation. This is also consistent with the observed increase of the total monomeric ACNs content as described before (Figure 1).

As shown in Figure 4, a drastic decrease in the concentration of each individual ACN was observed during the simulated intestinal phase. Comparing the three aglycons, Dp glucosides seem to be more sensitive to the alkaline environment. The relative reduction in the concentration of monoglucoside and diglucoside form was −80.1% and −91.1%, respectively. Pg and Cy glucosides underwent less degradation during the simulated intestinal phase (−49.1% and −57.6% for Pg-3-glucoside and Cy-3-glucoside and −12.9% and −5.5% for the respective diglucosides). It seems that the number of -OH groups in the B-ring affects the stability or the degradation rate of the monomeric ACNs. Thus, Dp glucosides, which possess three -OH groups in the B-ring, were reduced to a greater extent as compared to Pg and Cy glucosides, which possess one and two −OH groups, respectively. These findings are in accordance with previous research work performed in a digestive system [39,40]. It is worth noting that, in all cases under alkaline conditions, the percentage degradation of monoglucosides was greater (~63%) than the respective one of diglucosides (~38%). Furthermore, a statistically significant negative correlation (*R* = −0.91, *p* < 0.01) was found between the percent polymeric color and the sum of ACNs content during the SDP. 

### 3.3. Total Phenolic Content and Antioxidant Activity During the Simulated Digestion Process

During the gastric phase, the TPC remained practically constant (*p* > 0.05) (Table 2) and did not differ significantly from the TPC of undigested juice (1271 mg/L). A significant decrease in the TPC was observed during the shift from the gastric to the intestinal phase. This phase coincides with a change in pH to the alkaline region and with the evolution of the ACN degradation reactions. Moreover, TPC did not seem to be affected by the increase of the ellagic acid concentration, which raised from 1.6 mg/L at the beginning of the simulated gastric phase (0 h) to 9.2 mg/L at the beginning of the simulated intestinal phase (I-2 h).

We should note here that, from the chromatographic analysis of the non ACNs phenolic fraction, we were able to determine only the ellagic acid, although a large number of overlapping peaks was observed (results not shown). The concentration of ellagic acid reached the value of 11.2 mg/L by the end of SDP (I-6 h). On the other hand, the antioxidant activity of PJ did not significantly change throughout the SDP (*p* = 0.25).

## 4. Discussion

The major digestive segments in the human gastrointestinal tract include stomach and small intestine, where ACNs undergo modification mainly in response to changing pH values rather than to enzymatic action [11,29]. This is the reason that digestive enzymes were not included in the present study.

ACNs are a group of compounds of particular interest due to the health promoting properties attributed to them. Foods that contain ACNs, including pomegranate fruits and juice, are considered functional. The Mediterranean climate enhances the growth of good quality pomegranates and their juice is characterized by the higher values of antioxidant activity, TPC and total ACNs than those grown in a desert climate [41]. The PJ from the Ermioni variety, studied in this work, is characterized by moderate total monomeric ACN content, as compared to others grown in the Mediterranean. Particularly, the ACN content of nine registered Turkish pomegranate varieties ranged between 28 to 447 mg /L and it was strongly affected by the cultivar and the stage of maturity [35]. Similar findings have been reported by other investigators [4,37,41,42]. As reported by Todaro et al. (2016) [37], Cy is the predominant aglycon either as monoglucoside or as diglucoside. Cy-3-glucoside, which prevailed in the PJ of the Ermioni variety, is considered the most representative dietary ACN exhibiting a significant bioavailability as compared to the rest of the ACNs [10,20,21,43].

A high polymeric color can result from ACNs degradation as well as from the formation of a complex between ACNs and tannins [30]. The polymeric color is usually formed in juices obtained by pressing the whole pomegranates, due to their high content of condensed tannins that originate from the rind [35]. Since in our work, the juicy sacs from the fruit pericarp were separated by hand and pressed without the rinds, the PJ would probably have low amounts of condensed tannins. Our findings indicate that the increase of the polymeric color during the intestinal phase of digestion is mainly due to the ACNs degradation products rather than to the interaction between ACNs and condensed tannins, that could favor the formation of polymeric ACNs.

It has been reported that PJs, and more specifically the commercial ones, are characterized by threefold antioxidant activity than red wine and green tea infusion. The increased antioxidant activity of commercial PJ is attributed mainly to the rind tannins, which pass to the juice during the industrial extraction process [16]. More specifically, the antioxidant activity of PJ is highly attributable to its ellagitannin content, which is comprised of the gallagic acid, an analogue of ellagic acid containing four gallic acid residues, the punicalin, the principal monomeric ellagitannin in which gallagic acid is bound to glucose and punicalagin, a further ellagitannin in which ellagic acid is also linked to the glucose moiety. These ellagitannins, under the acidic conditions of the simulated gastric phase are hydrolyzed to give the free sugar moieties and ellagic acid as well as other phenolic acids [18]. However, in the present study, the higher amounts of ellagic acid were observed at the alkaline environment of the simulated intestinal phase. This discrepancy can be explained by considering the very low solubility of ellagic acid in aqueous acidic environment (gastric phase) as opposed to its high solubility in alkaline medium (intestinal phase) [44]. In pomegranate supplements, it was reported that the antioxidant activity and the TPC did not correlate with the presence of ellagic acid [44].

The increase of the total monomeric ACNs content of PJ during simulated gastric conditions is attributed to the fact that they are more stable at the acidic pH of the stomach where the form of red flavylium prevails. Under basic conditions (pH > 7), such as those occurring in the small intestine, the total monomeric ACNs content decreases as a result of the degradation of ACNs via the cleavage of the aromatic ring [18,29,43].

The evolution of the ACNs detected in the PJ of the Ermioni variety during the SDP followed the already mentioned pattern in the literature, namely that both the nature of the sugar conjugated and the number of –OH groups in the B ring affects their stability [45]. Pg and Cy based ACNs that possess one and two –OH groups, respectively, undergo less degradation during the simulated intestinal phase than Dp based ACNs with three –OH groups in their B-ring. McGhie et al. (2003) [19] studied the absorption of ACNs in vivo and also noticed a greater reduction of Dp based ACNs as compared with malvidin based ACNs. This was ascribed to the greater number of –OH groups in Dp which in turn enhance hydrophilicity. ACNs possessing more –OH groups on their aglycones are more susceptible to oxidation and thus to degradation [45]. The degradation of ACNs is spontaneous and usually leads to the formation of phenolic acids and aldehydes [43]. Particularly, Dp, Cy and Pg degrade to form gallic acid, protocatechuic acid, and 4-hydroxybenzoic acid, respectively, along with a common phenolic aldehyde, typically 2,4,6-trihydroxybenzaldehyde [39]. We can assume that the increase of the polymeric color observed at the end of the SDP was indirectly attributed not only to the degradation of ACNs but also in the formation of complexes between ACNs and these phenolic compounds. Moreover, the decrease of the total monomeric ACNs content and the constant antioxidant activity during SDP appeared to be contradictory. In order to explain this observation, we assumed that the phenolic acids deriving from the degradation of ACNs during the SDP presented similar antioxidant activity to the parent ACNs molecules. This is supported by other researchers [16] who reported similar antioxidant activity between Cy-3-glucoside and ellagic acid (both having four free phenolic –OH groups), whereas gallic acid presented twofold antioxidant activity, although it has three phenolic –OH groups.

During the SDP, it was found that about 65% of the total monomeric ACNs, of which 66% diglucosides and 37% monoglucosides, remained intact. There is evidence that ACNs are absorbed as intact glycosides [18] because they have been detected in plasma and urine in this form, both in animals and in humans [11]. ACNs with more hydrophilic groups exhibit superior bioavailability compared to ACNs with fewer hydroxyl groups and low hydrophilicity [21]. Thus, we can infer that the ACNs diglucosides are compounds with a higher biological activity than the corresponding monoglucosides. Therefore, the PJ of the Ermioni variety seems to retain a large amount of these bioactive compounds after the SDP.

Furthermore, the human microbiota possesses an enzymatic system that is able to metabolize ACNs and may play a major role in the production of compounds with different bioavailabilities and biological activities. As far as the food matrix is concerned, it seems to affect ACNs’ bioavailability [17]. Similar food matrices (i.e., carrots and carrot juice) do not significantly change the concentration of absorbed ACNs but they determine the rate of absorption [21]. Other researchers suggest that the consumption of even moderate doses of ACNs can lead to their accumulation in tissues. Therefore, research should be directed to the determination of the appropriate dose and the food matrix of which PJ may be part.

## Figures and Tables

**Table 1:** Chemical characteristics of pomegranate juice (PJ) from the Ermioni variety.

Parameter	Value
pH	3.3 ± 0.2
Total monomeric anthocyanins (mg/L) ^1^	187 ± 5
% Polymeric color	12.0 ± 1.2
Total phenolic content (mg/L) ^2^	1271 ± 40
EC_50_	0.35 ± 0.05
Anthocyanin Content (mg/L)	
Delphidin-3,5-diglucoside	54.5 (14.2%) ^3^
Cyanidin-3,5-diglucoside	104.3 (27.4%)
Pelargonidin-3,5-diglucoside	9.8 (2.4%)
Delphidin-3-glucoside	35.5 (9.2%)
Cyanidin-3-glucoside	155.3 (40.8%)
Pelargonidin-3-glucoside	23.4 (6.0%)
Total	382.8 ± 0.1

^1^ Determined by the pH-differential method. ^2^ Expressed as gallic acid equivalents. ^3^ Value in parenthesis represents the percent content of each individual anthocyanin relative to the total content of anthocyanins (ACNs) as determined using HPLC.

**Table 2:** Mean values of total phenolic content, antioxidant activity and ellagic acid concentration of pomegranate juice (Ermioni variety) during the simulated digestion process.

Sample ^1^	Total Phenolic Content (mg/L) ^2^	EC_50_ (Juice% *v/v*) ^3^	Ellagic Acid (mg/L)
0 h	1240 ± 43 ^A^	0.38 ± 0.05 ^A^	1.6 ± 0.1 ^A^
G-2 h	1248 ± 73 ^A^	0.39 ± 0.05 ^A^	1.6 ± 0.1 ^A^
G-4 h	1239 ± 54 ^A^	0.39 ± 0.05 ^A^	2.1 ± 0.3 ^AB^
G-6 h	1297 ± 39 ^A^	0.41 ± 0.09 ^A^	2.9 ± 0.3 ^B^
I-2 h	1086 ± 28 ^B^	0.43 ± 0.05 ^A^	9.2 ± 1.1 ^C^
I-4 h	1112 ± 18 ^B^	0.42 ± 0.05 ^A^	10.1 ± 1.8 ^C^
I-6 h	1086 ± 40 ^B^	0,43 ± 0,06 ^A^	11.2 ± 1.2 ^C^

^1^ 0h, G-2 h, G-4 h, G-6 h: samples under the gastric condition; I-2 h, I-4 h, I-6 h: samples under the intestinal condition. ^2^ expressed as gallic acid equivalents. ^3^ EC_50_: Efficient Concentration at 50% loss of DPPH activity. Different superscript letters denote statistical significance (*p* < 0.05).
